# Acute Psychotic Symptoms due to Benzydamine Hydrochloride Abuse with Alcohol

**DOI:** 10.1155/2014/290365

**Published:** 2014-09-29

**Authors:** Yahya Ayhan Acar, Mustafa Kalkan, Rıdvan Çetin, Erdem Çevik, Orhan Çınar

**Affiliations:** ^1^Department of Emergency Medicine, Etimesgut Military Hospital, Etimesgut Asker Hastanesi, Etimesgut, 06797 Ankara, Turkey; ^2^School of Medicine, Gulhane Military Medical Academy, GATA Tıp Fakültesi, Etlik, 06010 Ankara, Turkey; ^3^Department of Emergency Medicine, Van Military Hospital, Van Askeri Hastanesi, Altıntepe, 65040 Van, Turkey; ^4^Department of Emergency Medicine, Gulhane Military Medical Academy, GATA Acil Tıp AD, Etlik, 06010 Ankara, Turkey

## Abstract

Benzydamine hydrochloride is a locally acting nonsteroidal anti-inflammatory drug. Benzydamine hydrochloride overdose can cause stimulation of central nervous system, hallucinations, and psychosis. We presented a young man with psychotic symptoms due to benzydamine hydrochloride abuse. He received a total dose of 1000 mg benzydamine hydrochloride with alcohol for its hallucinative effects. Misuse of benzydamine hydrochloride must be considered in differential diagnosis of first-episode psychosis and physicians should consider possibility of abuse in prescribing.

## 1. Introduction

Benzydamine hydrochloride (BH) is a locally acting nonsteroidal anti-inflammatory drug (NSAID) with local anesthetic and analgesic properties for pain relief [1]. BH is available in capsule form, mouthwash, dermal cream, aerosol, and vaginal douche preparations. Proprietary names of BH are Tantum (in Austria, Canada, Germany, Italy, The Netherlands, South Africa, Spain, Switzerland, and Turkey), Difflam (in the United Kingdom, Australia), and Opalgyne (in France) [2].

Its reported side effects are urticaria, erythema, rash, photosensitivity, bronchospasm, and renal dysfunction. BH's daily maximum oral usage dose is 200 mg/day. Hallucinations, stimulation of central nervous system, excitation, hyperactivity, paranoia, dry mouth, and convulsions may occur in oral dosages of 500–3000 mg. Recreational use of benzydamine is popular in Poland and Brazil and recently some cases were reported from Turkey also [3, 4].

In this case report we present a young man that received high dose BH with alcohol and associated psychotic symptoms.

## 2. Case Report

A twenty-year-old man was admitted to emergency department with the complaint of acute visual hallucinations of seeing bugs. It was learned that he had received twenty capsules of Tantum (50 mg BH) orally (total dose of 1000 mg) with alcohol last night. However, it was thought that it was a suicidal attempt at first sight; then it was learned that he had misused BH with alcohol for its hallucinative effects. He expressed that he had learned this effect of BH from internet forum sites and bought the drug without prescription and that was the first misuse attempt of him. He denied any prior psychiatric disorder.

His vital signs were in normal ranges (BP: 120/70 mmHg, pulse rate: 80/bpm, SaO_2_: 96%, and body temperature: 36.7°C) and any neurologic deficit was not detected. Cognitive examination did not show any abnormality. In laboratory examination, ECG was normal sinus rhythm and there was not any abnormality in blood gas analysis, complete blood count, and routine biochemical measurements. Breathalyzer test was negative. Urine or blood drug screen was not obtained.

The patient was referred to the psychiatry clinic for further evaluation and he was discharged without prescription within the same day. Patient could not be reached to ascertain whether the condition was resolved and there was not any record on further psychiatry clinic admission.

## 3. Discussion

BH preparates are over-the-counter in many countries and misuse reports are increasing [5, 6]. In Turkey, BH preparates could be sold without prescription but Ministry of Health of Turkey prohibited selling BH preparates without prescription in 2012 after increased number of abusing cases. However, as seen in this case, the precautions seem not to be sufficient to prevent selling BH without prescription.

Hallucinations were reported as a frequent symptom after ingestion of benzydamine-containing vaginal preparations [5]. Doğan et al. reported a case report of visual hallucinations after BH overdose (total dose of 250 mg, 14.7 mg/kg) for suicide in a 11-year-old girl [4]. Our case was an adult man and took a total dose of 1000 mg (14.2 mg/kg) BH with alcohol for hallucinative effects.

All of the reported cases received BH orally as our case. Usage with alcohol is one of the reported ways of abusing BH and our case was also abusing BH with alcohol [3]. However it is hard to differentiate hallucinations from alcoholic hallucinosis; our patient was not an alcoholic and had visual hallucinations rather than auditory hallucinations.

Normal dose of BH may cause psychiatric side effects in patients who have psychiatric disorders, and overdose may result in chronic psychosis [7, 8]. Our patient denied any prior psychiatric disorder.

BH is a widely used NSAID and psychotic symptoms were reported after abuse or overdose but there is no clear evidence how BH causes hallucinations mechanistically. These transient symptoms may result in fatal complications or chronic disorders. Physicians should be aware of psychotic adverse effects of BH and keep in mind these side effects while prescribing and evaluating patient especially patients with first-episode psychosis.
